# Adjuvant Drug-Assisted Bone Healing: Advances and Challenges in Drug Delivery Approaches

**DOI:** 10.3390/pharmaceutics12050428

**Published:** 2020-05-06

**Authors:** Rebecca Rothe, Sandra Hauser, Christin Neuber, Markus Laube, Sabine Schulze, Stefan Rammelt, Jens Pietzsch

**Affiliations:** 1 Department of Radiopharmaceutical and Chemical Biology, Institute of Radiopharmaceutical Cancer Research, Helmholtz-Zentrum Dresden-Rossendorf (HZDR), 01328 Dresden, Germany; r.rothe@hzdr.de (R.R.); s.hauser@hzdr.de (S.H.); c.neuber@hzdr.de (C.N.); m.laube@hzdr.de (M.L.); 2 School of Science, Faculty of Chemistry and Food Chemistry, Technische Universität Dresden, 01069 Dresden, Germany; 3 University Center of Orthopaedics and Traumatology (OUC), University Hospital Carl Gustav Carus, 01307 Dresden, Germany; Sabine.Schulze1@mailbox.tu-dresden.de (S.S.); stefan.rammelt@uniklinikum-dresden.de (S.R.); 4 Center for Translational Bone, Joint and Soft Tissue Research, University Hospital Carl Gustav Carus and Faculty of Medicine, Technische Universität Dresden, 01307 Dresden, Germany; 5 Center for Regenerative Therapies Dresden (CRTD), Tatzberg 4, 01307 Dresden, Germany

**Keywords:** angiogenesis, bioactive scaffolds, bone grafting, critical-size bone defects, drugs, inflammation, tissue regeneration, osteoconduction, osteoinduction, osseointegration

## Abstract

Bone defects of critical size after compound fractures, infections, or tumor resections are a challenge in treatment. Particularly, this applies to bone defects in patients with impaired bone healing due to frequently occurring metabolic diseases (above all diabetes mellitus and osteoporosis), chronic inflammation, and cancer. Adjuvant therapeutic agents such as recombinant growth factors, lipid mediators, antibiotics, antiphlogistics, and proangiogenics as well as other promising anti-resorptive and anabolic molecules contribute to improving bone healing in these disorders, especially when they are released in a targeted and controlled manner during crucial bone healing phases. In this regard, the development of smart biocompatible and biostable polymers such as implant coatings, scaffolds, or particle-based materials for drug release is crucial. Innovative chemical, physico- and biochemical approaches for controlled tailor-made degradation or the stimulus-responsive release of substances from these materials, and more, are advantageous. In this review, we discuss current developments, progress, but also pitfalls and setbacks of such approaches in supporting or controlling bone healing. The focus is on the critical evaluation of recent preclinical studies investigating different carrier systems, dual- or co-delivery systems as well as triggered- or targeted delivery systems for release of a panoply of drugs.

## 1. Introduction

Bone defects regularly result after trauma, tumor resection, or infection and are becoming an increasing challenge with a rising number of elderly patients with relevant comorbidities. Because of an ageing population due to an increased life expectancy in the industrial countries, pathologic fractures and other bone-related manifestations of systemic diseases are becoming more frequent. The structure of adult and particularly ageing bone is more fibrous and comprises fewer bone-forming cells compared to children, leading to a reduced repair capacity. Besides comorbidities and age, gender represents another risk factor for impaired fracture healing. Ageing women are more frequently affected by osteoporosis in comparison to men based on their hormonal status. Estrogen deficiency reduces bone mineral density, making the bone more fragile. If bone defects are of critical size or patients have an impaired bone healing due to metabolic or inflammatory diseases such as diabetes mellitus and osteoporosis, the defects will not heal completely without additional surgical intervention [1,2,3,4,5,6].

Bone, as a complex structured tissue, requires a well-orchestrated healing process regarding timeframe and localization. In the literature, the bone healing process and defining molecular pathways are already described extensively [2,3,6,7,8,9,10,11,12,13]. Briefly, bone regeneration can be sectioned into an inflammatory, reparative, and remodeling phase. Within the several repair stages, distinct cell populations, such as mesenchymal stem cells, immune cells, or endothelial cells together with bone-specific osteoblasts and osteoclasts interact within a complex network and occupy the leading role in alternating turns. The cells release diverse growth factors and signaling mediators to stimulate vascularization and matrix formation resulting in regenerated bone tissue and reestablished normoxic conditions [14].

Currently, the “gold standard” for therapeutic intervention in long bone defects are bone autografts in combination with stable internal or external fixation. Autografts belong to the first generation of bone substitutes, but have some drawbacks, including reduced bioactivity, limited availability, and donor site morbidity. Internal or external fixation may fail in patients with impaired bone healing and prolonged offloading and immobilization will further add to the overall morbidity. Therefore, intensive research has focused on new bioactive materials being able to control the host tissue reaction and include tissue engineering aspects. According to latest expertise, the optimal bone scaffold should combine osteoconductive and osteoinductive properties to facilitate osteogenesis. This “diamond concept” demands the interconnection between three-dimensional scaffolds for mechanical support, growth factors, or other bioactive molecules, and osteogenic cells. Although intensive research has been carried out on these concepts, the gap towards translation into clinical application has yet to be closed. Thus, there is an urgent clinical need for smart adjuvant therapy approaches with bone scaffolds being biocompatible, well implantable in host tissue, and suitable as drug delivery platforms [1,2,3,7,8,9,15,16,17].

The following review examines carrier systems and dual delivery systems for release of targeted adjuvant therapies to enhance bone regeneration using inflammation-modulatory agents, pro-angiogenic agents, and metabolism-modulatory compounds (Table 1). To ensure optimal bone fracture treatment, the administered dose, as well as chemical properties of the incorporated drugs and release mechanisms, have to be taken into account when fabricating and establishing drug delivery systems [18,19]. In the following sections, several aspects regarding composition and formulations of drug carrier, and drug release kinetics due to diffusion, scaffold degradation, or triggered release will be highlighted.

A PubMed database search was performed in March 2020 using key words and phrases “drug delivery”, “local”, “scaffold”, “inflammation”, “angiogenesis”, “trigger”, “release kinetic”, linked to the key words “bone defect”, “fracture”, and “healing” by AND/OR as Boolean function.

## 2. Carriers for Drug Delivery

**Figure 1:**
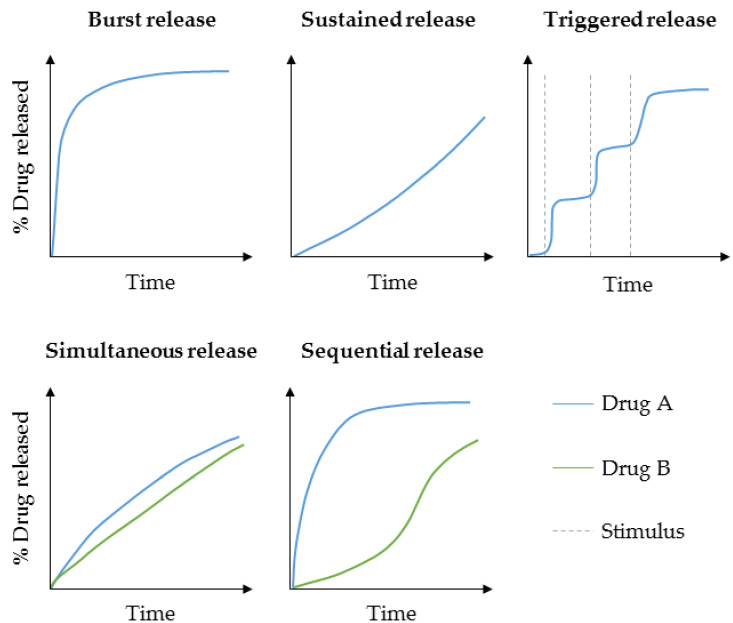
Exemplary schematic drug release kinetics for single and dual drug delivery.

Targeted drug delivery approaches are needed to increase the therapeutic drug efficacy and decrease adverse side effects [6,23,24,25]. Ideal scaffolds should fulfill several biological and structural requirements including biocompatibility, biomimicry, mechanical support, and porosity to permit cell adhesion as well as nutrient supply, biodegradability after successful therapy, and controlled drug delivery [1,13,17,26,27,28,29,30,31]. Regarding optimal scaffold loading with desired drugs, several aspects such as loading capacity, distribution, binding affinities, and stability have to be considered [32]. In general, drug release kinetics (Figure 1) can be tuned by drug loading techniques, scaffold morphology, and degradation according to polymer composition, crosslinking degree, or porosity [33].

### 2.1. Composition of Scaffolds

Several reviews have summarized a variety of in vivo studies regarding various natural, synthetic, or inorganic drug delivery approaches and their bone regenerative potential as well as drug release kinetics [13,34,35,36,37,38,39].

#### 2.1.1. Natural Polymers

**Figure 2:**
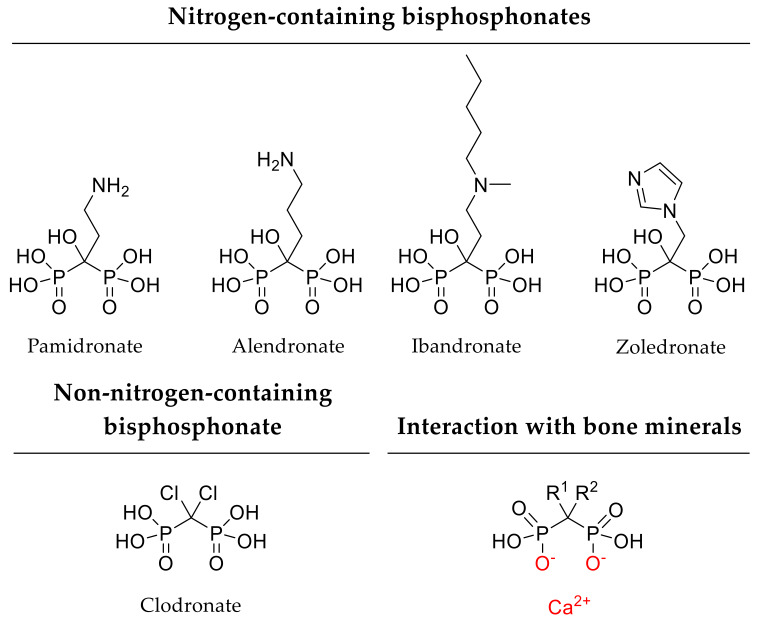
Chemical structures of selected anti-resorptive bisphosphonates. Bisphosphonates improve the bone quality due to accelerated bone mineralization and are frequently used in the treatment of osteoporosis. The hydrophilic molecules contain the typical P–C–P core structure and exhibit a high binding affinity to bone minerals.

Natural scaffolds are highly biocompatible as they belong to the extracellular matrix (ECM) in most cases, but feature only minor mechanical strength and can lead to undesired immune reactions. The most studied natural polymers comprise collagen, hyaluronic acid, alginate, and chitosan [13,29,33,34,36,40]. In a clinical trial with a study length of 12 months, rhBMP-2 (recombinant human bone morphogenetic protein 2, 1.50 mg/mL, 12 mg total dose) delivery from an absorbable collagen sponge accelerated the bone healing of tibial fractures. Significantly, the drug delivery approach reduced the amount of secondary interventions and infections compared to the control group without the BMP-2-delivering implant [41]. Aoki and Saito reviewed several natural polymers including collagen, gelatin, and hyaluronic acid with respect to BMP-2 delivery and the effects on bone healing [42]. Furthermore, Guo and coworkers investigated a drug delivery system comprising collagen sponges loaded with ibandronate (Figure 2). In vitro, release of this anti-resorptive bisphosphonate from collagen proceeds rapidly over a few days. After 4 weeks, local ibandronate (0.04 mg/10 mg scaffold) delivery improved bone healing and mechanical properties in an osteoporotic rat femoral fracture model as the treatment resulted in an enhanced callus formation with higher density compared to control groups without scaffolds, unloaded collagen sponges or implants combined with systemic ibandronate administration [43]. Therefore, a smart drug delivery system for bone regenerative applications should include natural polymers to ensure biocompatibility and provide the basis for osteogenesis due to facilitated cell adhesion and migration.

#### 2.1.2. Calcium Phosphates

Calcium phosphates are suitable scaffolds in bone tissue regenerative applications as these matrices have desirable mechanical strength and can be easily functionalized or adapted to the diamond concept becoming efficient bioactive drug delivery systems [33,34,44,45,46]. According to the literature, the most common bone tissue engineering devices comprise calcium phosphates including hydroxyapatite and composites [17,29,33,36,47,48,49]. Calcium phosphates can be applied in form of granules, ceramics, or cements [50]. Several reviews summarized several in vivo studies dealing with hydroxyapatite- and calcium phosphate-based materials for site-directed delivery of growth factors including BMP-2, VEGF (vascular endothelial growth factor), or TGF-ß (transforming growth factor-ß) [50,51]. For instance, an investigation by Poldervaart and coworkers focused on an alginate hydrogel with incorporated BMP-2-loaded gelatin microparticles and calcium phosphate granules. In vitro, BMP-2 release kinetic showed a characteristic initial burst followed by a degradation-dependent release due to the presence of proteolytic enzymes such as collagenases. BMP-2 delivery by this cylindrical calcium phosphate scaffold increased ectopic bone formation in rats after 12 weeks [52]. Likewise, Chu and coworkers incorporated BMP-2 (10 µg/scaffold) in calcium phosphate-based scaffolds and found improved mechanical properties, like stiffness of bone, in rat femoral defects after an investigation period of 15 weeks [53]. Moreover, Maehara and coworkers used hydroxyapatite-collagen sponges for FGF-2 (fibroblast growth factor 2, 10–100 µg/mL) delivery in rabbit femoral trochlear groove defects. In vivo, growth factor-loaded scaffolds tended to improve bone regeneration, whereas even the empty scaffold itself elicited sufficient bone repair properties up to 24 weeks [54]. Similarly, Komaki and coworkers used a drug delivery system comprising tricalcium phosphate and collagen for local release of FGF-2 (200 µg/0.3 mL scaffold). In vivo, the authors showed enhanced bone formation based on their FGF-2 delivery approach 12 weeks after implantation in rabbit tibial defects compared to unloaded controls [55]. The same scaffold composition of tricalcium phosphate and collagen is also suitable for PDGF (platelet-derived growth factor) delivery as shown in another study. The authors found advantageous effects regarding bone fracture healing due to local application of their drug delivery system without taking a closer look at the release kinetics in a diabetic rat model simulating an impaired fracture healing. Eight weeks after implantation, drug delivery systems containing 22 µg PDGF per scaffold increased mechanical strength [56]. Besides osteogenic growth factors, calcium phosphates are also appropriate scaffolds for delivery of angiogenic agents. Wernike and coworkers investigated the release kinetics of VEGF from calcium phosphate ceramics. The authors analyzed two different drug-loading techniques and adsorbed or co-precipitated VEGF, whereas co-precipitation of the growth factor reduced initial burst characteristics in comparison to adsorption. In vivo, sustained VEGF delivery from this calcium phosphate scaffold supported bone formation and vascularization in a murine critical-size cranial defect model after 28 days [57].

**Figure 3:**
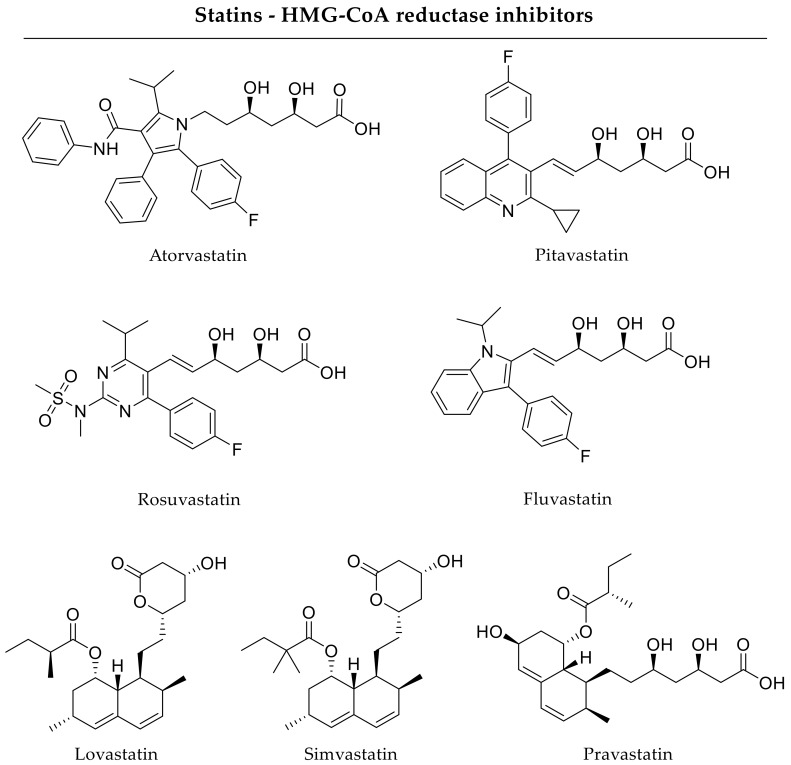
Chemical structures of selected HMG-CoA reductase inhibitors. The statins elicit pleiotropic effects regarding bone regeneration, despite having different carbo- and heterocyclic core structures. Anabolic impacts rely on stimulation of osteogenesis, but the statins also evoke dose-dependent anti-inflammatory and pro-angiogenic effects.

Furthermore, incorporation of adjuvant drugs in scaffolds for bone regenerative applications is required to ensure optimal fracture healing. By the use of simvastatin-loaded calcium sulfate scaffolds, Huang and coworkers indicated accelerated bone formation in a rabbit ulnar defect model. Regarding release kinetics of the HMG-CoA reductase inhibitor simvastatin (Figure 3), over 70% of the drug was released within 2 weeks, with a higher simvastatin loading resulting in a reduced release rate. In vivo, simvastatin-loaded scaffolds promoted bone regeneration in the same magnitude as calcium sulfate scaffolds loaded with BMP-2 resulting in similar newly formed bone tissue areas up to 8 weeks after implantation [58]. In addition, Nyan and coworkers investigated simvastatin (1 mg/scaffold) delivery by calcium sulfate discs and showed an improved bone formation in a rat critical-size calvarial defect model after 8 weeks compared to untreated controls or unloaded scaffolds [59]. In in vitro investigations, Khurana and coworkers analyzed pitavastatin-loaded injectable calcium phosphate foams. The foams released pitavastatin (Figure 3) by an initial burst. In vitro, pitavastatin delivery increased BMP-2 and VEGF expression indicating advantageous osteogenic and angiogenic effects [60]. Furthermore, pravastatin (Figure 3) can be used in bone regenerative applications as well. Delivery approaches based on local release forms of pravastatin have not been studied comprehensively yet, but oral administration in ovariectomized rats prevented bone loss indicating advantageous effects on bone metabolism [61]. In general, statins elicit pleiotropic effects [62]. Of note, statins inhibit HMG-CoA reductase, which is the rate-limiting enzyme in the mevalonate pathway responsible for the production of non-sterol and sterol isoprenoids, especially cholesterol. Thus, they became prominent lipid-lowering agents for the treatment of hypercholesterolemia [63]. In addition, statins can activate the AKT1/PI3K (protein kinase B/phosphatidylinositol 3-kinase) signaling pathway, which leads to some similar downstream effects as the inhibition of HMG-CoA reductase. The mevalonate pathway, on the other hand, can also be inhibited at other sites, such as the conversion of dimethylallyl pyrophosphate to geranyl pyrophosphate by bisphosphonates. This association points to another possible pleiotropic effect of statins. In fact, the statins have gained attraction as a pro-osteogenic molecule after first indications that lovastatin (Figure 3) can stimulate the production of important osteogenic growth factors [64].

By investigating bone healing of rat tibial defects over 8 weeks, Park and coworkers found increased bone formation and mineralization after application of alendronate-loaded calcium phosphate scaffolds. In vitro, alendronate (Figure 2) displayed a sustained release over 28 days. Thereby, scaffolds incorporating high alendronate concentration (5 mg/scaffold) had a diminished release profile as only 20% of the drug was released after 4 weeks in comparison to lower alendronate concentration (1 mg/scaffold), where 70% of the bisphosphonate were released [65]. Apart from osteogenesis, implant loosening often remains challenging and drug delivery approaches should address this issue. According to several in vivo studies, strontium-modified calcium phosphate implants support osseointegration and local bone formation in a rat osteoporotic femoral defect model over an investigation period of 6 weeks and 6 months [66,67]. For instance, Tao and coworkers focused on a drug delivery system composed of strontium-modified calcium phosphate cement and an additional single local administration of BMP-2 (5 µg/mL). In osteoporotic rats, the strontium-containing scaffold as well as the combination of this scaffold with BMP-2 led to an increased bone formation of critical-size femoral defects after 8 weeks [68]. For similar investigation of strontium-doped calcium phosphate cements, Reitmaier and coworkers used critical-size bone defects in sheep. Local strontium release resulted in enhanced bone formation after 6 months, but not after short-term treatment [69]. Consequently, calcium phosphates meet several aspects of the diamond concept for ideal bone implants as these scaffolds provide structural support and feasibility of incorporating a variety of different adjuvant drugs.

#### 2.1.3. Synthetic Polymers

In contrast to scaffolds based on natural compounds, synthetic polymers have a clear defined chemistry and can be easily processed and modified in terms of mechanical strength and biodegradability. Nevertheless, synthetic polymers often lack bioactive effects and, therefore, are often combined with osteoinductive drugs. The most studied synthetic polymers include polyethylene glycol (PEG), polycaprolactone (PCL), polylactic acid (PLA), polylactide-co-glycolide (PLGA), and their derivatives [3,29,33,34,40,70,71]. For instance, Li and coworkers incorporated a prostaglandin E2 agonist (0.05–5 mg/scaffold) into a PLGA-derived matrix. According to the authors, release of the prostaglandin E2 receptor selective drug proceeded over 14 days and improved bone properties regarding callus size, density and strength in a rat femoral fracture model after 21 days [72]. Yoshii and coworkers investigated lovastatin-loaded polyurethane scaffolds in a rat femoral defect model. Lovastatin showed slow and sustained release kinetics with an almost linear trend. In vivo, an improved bone density after local drug release was visible after 4 weeks [73]. Without determining release kinetics, Kaigler and coworkers detected advantageous effects regarding angiogenesis and osteogenesis 12 weeks after implantation of VEGF-loaded PLGA scaffolds in rat calvarial defects [74]. By using fluvastatin-loaded PLGA membranes, Zhang and coworkers were able to show enhanced bone formation in both a rat calvarial and tibial defect model after 4 and 8 weeks. According to the authors, the PLGA membranes released fluvastatin with a release rate of approximately 1 µg/day [75]. Nevertheless, designed smart scaffolds potentially ought to comprise both natural and synthetic polymers to fulfill all requirements.

#### 2.1.4. Hybrid Scaffolds

To combine tunable scaffold characteristics based on synthetic polymers and biocompatibility of natural compounds, hybrid materials could be very promising drug delivery systems. Dang and coworkers established a PTH (parathyroid hormone) delivery system comprising alternating stacked polyanhydride isolation layers and drug-loaded alginate layers. This fabricated scaffold is supposed to release PTH in a pulsatile manner over 21 days as intermittent PTH release is favorable for bone repair. Modulation of chemical composition and thickness of the isolation layer resulted in the desired daily pulsatile release profile. In vivo, the programmed PTH release enhanced bone regeneration in a murine calvarial defect model after 3 or 8 weeks without affecting bone mineral density [76,77]. Jeon and coworkers made use of another anabolic drug, BMP-2 (1 µg/scaffold), and compared drug release kinetics and bone healing potential of heparin-conjugated PLGA scaffolds with unconjugated polymeric scaffolds. Both, hybrid and synthetic scaffold were loaded with BMP-2 and investigated in a rat ectopic bone formation model. Regarding BMP-2 release, unconjugated scaffolds released the total amount of incorporated BMP-2 within 4 h, whereas the heparin-conjugation sustained BMP-2 release at least over 2 weeks. In vivo, BMP-2-loaded hybrid scaffolds resulted in the greatest bone regeneration in comparison to unloaded or unconjugated PLGA scaffolds after 8 weeks [78]. Additionally, Kim and coworkers also made use of heparin-functionalized scaffolds. The authors conjugated PCL-PLGA scaffolds with heparin-dopamine to achieve a controllable release of the immobilized BMP-2. In vitro, BMP-2 release exhibits burst release kinetics within the first hours followed by lower and sustained release over 2–3 weeks. In a rat femoral defect model, BMP-2 delivery by the conjugated synthetic scaffold increased bone and callus formation 8 weeks after implantation in comparison to unloaded scaffolds and scaffolds without dopamine conjugate [79]. Hoshino and coworkers studied another hybrid scaffold formulation over 12 weeks. The authors used a scaffold consisting of tricalcium phosphate and a PLA-PEG polymer for delivery of BMP-2 (80 µg/implant). By investigation of rib defects in dogs, the authors showed an improved bone regeneration and stiffness after application of BMP-2-loaded hybrid scaffolds in comparison to BMP-2-loaded tricalcium phosphate alone [80]. By using PLGA-coated gelatin sponges, Kokubo and coworkers showed enhanced bone repair in critical-size rabbit ulnar defects after BMP-2 delivery resulting in a complete union after 16 weeks [81]. Keskin and coworkers fabricated a BMP-2 delivery system based on collagen-chondroitin sulfate discs, which further contained a polymeric coating. Regarding BMP-2 delivery, the drug carrier released about half of the incorporated growth factor within 2 weeks. In a rat femoral defect model, the authors found osteoinductive effects in response to short-term BMP-2 delivery over 3 weeks by histological investigation [82]. Reichert and coworkers used a hybrid scaffold consisting of PCL and tricalcium phosphate for local BMP-7 delivery. In critical-size tibial defects of sheep, the drug delivery approach increased bone volume and mechanical strength after application of their BMP-7 delivery approach in comparison to unloaded scaffolds resulting in bridging after 3 months and increased strength after 12 months [83]. Moreover, other investigations dealt with a hybrid membrane consisting of chitosan and silica components for local delivery of adsorbed BMP-2 in terms of release kinetics and bone regeneration of calvarial defects. In vitro, BMP-2 exhibits sustained release kinetics, whereas the initial release of BMP-2 from the hybrid membrane at the first day was higher in comparison to BMP-2-loaded chitosan membranes. After 2 weeks, loaded hybrid membranes accelerated bone regeneration by almost a third in a rat model compared to unloaded hybrid scaffolds [84].

Besides anabolic growth factors, osteogenic and angiogenic drugs have also been coupled with hybrid drug carriers. Piskin and coworkers investigated two different loading methods of PCL nanofibers forming membranes and their bone regenerative potential. The authors compared PCL scaffolds having simvastatin absorbed to the surface with scaffolds, where simvastatin was incorporated into the nanofibers within the fabrication process. Scaffold loading with simvastatin based on commercially available simvastatin tablets for oral use containing also cellulose, starch, and other inactive components. The loading method mentioned first led to a burst release, whereas the direct incorporation of simvastatin into the nanofibers resulted in a sustained release profile according to the authors. In rat cranial bone defects, drug delivery by PCL scaffolds with direct incorporation of simvastatin enhanced bone mineralization in comparison to the absorptive loading after 6 months [85]. Concerning statin delivery to bone defects, Ibrahim and Fahmy investigated different chitosan-based sponges for local rosuvastatin delivery (Figure 3) over 4 weeks. The authors used polyacrylic acid or polyacrylic acid crosslinked with divinyl glycol (‘polycarbophil’) as anionic polymers. Variation of chitosan and polymer ratio modulated in vitro release kinetics, whereas rosuvastatin release was completed within a few hours reflecting burst release kinetics. The authors found signs of bone remodeling in histological investigations of rat femoral fractures due to local rosuvastatin delivery [86]. Similarly, Monjo and coworkers incorporated rosuvastatin in a collagen sponge. For stabilization of the collagen sponges, the authors used titanium implants with Teflon caps. This drug delivery approach released rosuvastatin through a burst, but did not change bone volume in a critical-size tibial defect in rabbits significantly after 4 weeks [87].

In summary, a combination of synthetic and natural compounds within the drug delivery scaffold could improve the bone formation efficacy.

### 2.2. Scaffold Formulations

**Figure 4:**
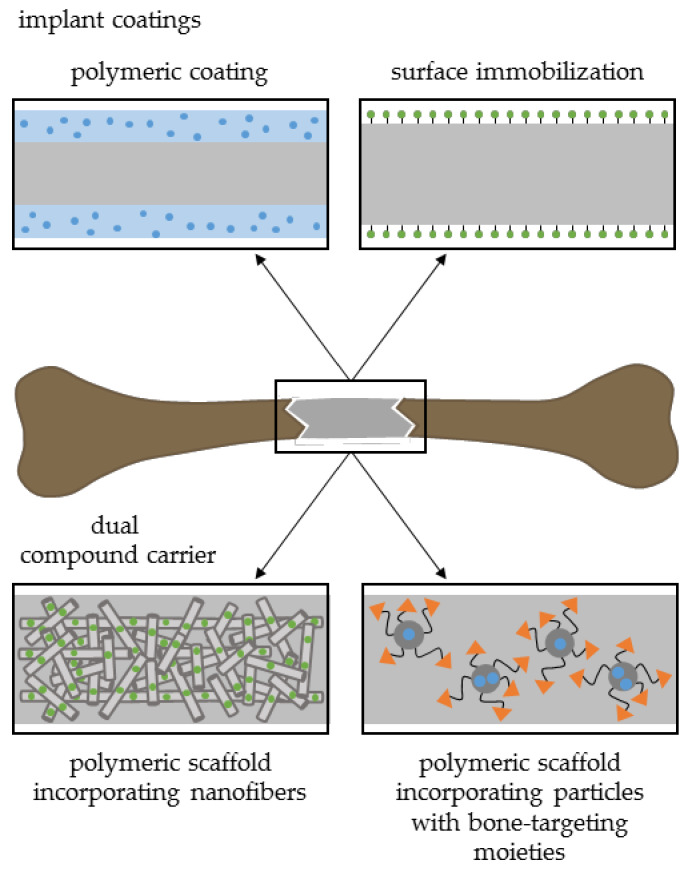
Exemplary scaffold formulations for bone-targeted drug delivery.

Fabricated drug delivery approaches originating from the above-mentioned polymeric components exist in various forms or shapes with different drug release profiles. Scaffold shape, morphology, porosity, molecular weight, or size, among others, can affect drug delivery decisively [88]. Nyberg and coworkers summarized several drug-eluting technologies such as bulk incorporation, surface adsorption, multilayer coatings, or particles (Figure 4) with regard to their advantages and disadvantages [89]. In the following section, various aspects of drug release kinetics of diverse scaffold formulations will be discussed in detail.

#### 2.2.1. Drug-Releasing Coatings

In bone regenerative applications, implant coatings can contain non-releasable and releasable components. To strengthen bone integration of implants, osteoconductive coatings including calcium phosphate or hydroxyapatite showed promising results. To date, several reviews have summarized techniques to apply osteoconductive coatings on implants with their related advantages and disadvantages [47,90,91]. In addition, non-fouling coatings for infection prevention and bioadhesive coatings consisting of ECM proteins are also important aspects in terms of improving bone fracture healing. Within this surface functionalization, peptide-binding sequences such as RGD (arginine-glycine-aspartate) or other integrin-binding domains were adsorbed on implant surfaces for mediation of cell adhesion and differentiation. As implant coatings seem to be attractive strategies in the field of bone healing, adsorption of bioactive drugs appear to be interesting in particular [3]. Subsequently, several drug-releasing coatings will be described, whereas relevant features of non-releasing coatings outlined above will be not considered in more detail.

**Figure 5:**
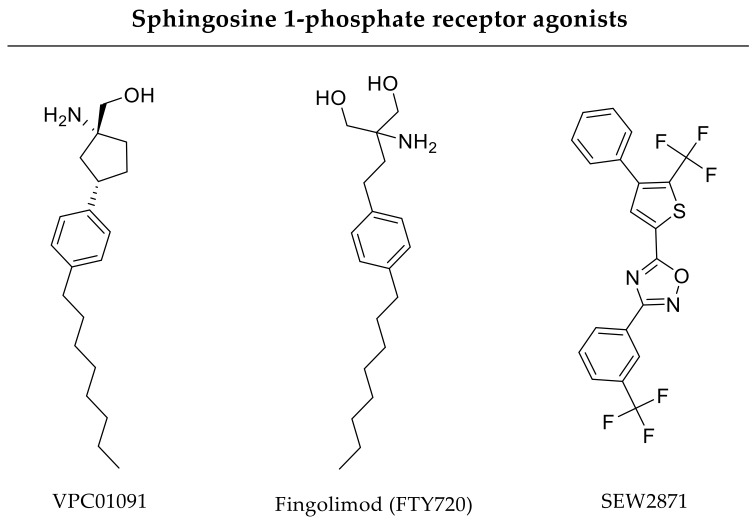
Chemical structures of sphingosine 1-phosphate receptor agonists. The lipid mediators act mainly on vascularization and bone metabolic cells.

Implant functionalization with immobilized growth factors or other drugs could be promising regarding enhanced bone healing, but several issues have to be taken into consideration while establishing drug-eluting coatings. For instance, immobilization might influence the biological activity of the surface-immobilized osteoinductive molecules depending on their orientation. Moreover, surface adsorption often causes unregulated release kinetics such as an initial burst [3]. Nevertheless, Petrie Aronin and coworkers coated bone allografts with a PLGA polymer containing FTY720 (Figure 5). This sphingosine 1-phosphate receptor agonist is supposed to be released locally with sustained release kinetics. In vitro, the authors measured an initial burst release within 5 days, probably due to degradation of the polymer coating, and a complete elution of FTY720 within 2 weeks. In a critical-size tibial defect model, FTY720 release improved mechanical stability compared to uncoated or unloaded polymer coatings 6 weeks after implantation [92]. Likewise, Das and coworkers used FTY720 incorporated within polymer-coated allografts to improve bone formation of critical-size tibial defects after 8 weeks [93]. In cranial defect models, Wang and coworkers, as well as Huang and coworkers, proved the bone regenerative effects of bone allografts coated with FTY720 after 12 and 8 weeks, respectively, whereby FTY720 release followed a characteristically initial burst and prolonged release kinetics, hereinafter [94,95].

To accelerate calvarial bone regeneration, Ishack and coworkers analyzed drug-releasing collagen coatings of hydroxyapatite-calcium phosphate scaffolds. The coatings contained either dipyridamole (100 µM) to raise local adenosine level and stimulate adenosine receptor-mediated downstream signaling or BMP-2 (200 ng/mL) as a well-known osteoinductive growth factor. Ex vivo, the authors were able to identify a sustained release of dipyridamole over an investigation period of 240 h. In vivo, this drug delivery system improved bone regeneration for both drugs in comparison to control scaffolds after 8 weeks [96]. By modifying the coating with respect to the amount of polymer and incorporated drug concentration, which affects coating thickness and BMP-2 distribution, respectively, drug release kinetics can be tuned from an initial burst towards a sustained release. The sustained release over 4 weeks slightly shifted improved bone formation and mineralization towards later bone healing phases in comparison to an initial burst release during the first 2 days [97,98]. To combat burst release, Shah and coworkers investigated drug-releasing implant coatings by using a layer-by-layer fabrication approach. Implant coatings consisted of an osteoconductive base layer with hydroxyapatite and chitosan combined with osteoinductive BMP-2-containing layers on top. By applying several layers, the drug release was modulated with respect to hydrolytic degradation of the polymeric layers. In this study, the authors followed up BMP-2 release in vivo and found a controlled growth factor release over 4 weeks, suggesting that a layer-by-layer coating technique prolongs drug delivery in comparison to simple surface adsorption or other drug-containing coatings with burst release kinetics [99]. Regarding the layer-by-layer technique, drug release rate can be tuned easily based on drug concentration and number of applied layers to combat drug diffusion. Nevertheless, to achieve a controlled release, many layers have to be fabricated [100]. Furthermore, He and coworkers implanted titanium alloys coated with hydroxyapatite-collagen composites into dog femora for 4 weeks. The implant coating contained BMP-2 for local drug delivery. In vivo, the BMP-2-loaded implants improved bone formation by almost a third compared to unloaded hydroxyapatite-coated alloys [101]. Moreover, Pauly and coworkers compared the bone regenerative effects of simvastatin-coated titanium implants with BMP-2-coated scaffolds, representing a positive control, after 42 days. The authors showed an improved tibial fracture healing in rats based on simvastatin delivery in high concentrations (50 µg/implant), which elicited a comparable bone regeneration of local BMP-2 (50 µg/implant) delivery [102].

In terms of optimized implant fixation, Li and coworkers investigated hydroxyapatite-coated titanium implants and implants with strontium-substituted coatings (10% strontium-hydroxyapatite). Strontium-containing implants improved osseointegration in osteoporotic rat tibia after 12 weeks [103]. Another approach to oppose implant loosening involves bisphosphonate delivery. Gao and coworkers investigated the potential of hydroxyapatite-coated titanium implants with immobilized bisphosphonates such as pamidronate, ibandronate, and zoledronate (1 mg/mL, Figure 2). In vitro, bisphosphonate release generally showed a burst release within the first 3 days and a subsequent slower discharge up to 7 days. With respect to the release kinetics, differences between the three bisphosphonates became obvious, as zoledronate was released fastest, followed by ibandronate and pamidronate. According to the authors, the different affinities of the three bisphosphonates to hydroxyapatite were responsible their individual release kinetics. In an osteoporotic rat model, the examined bisphosphonates improved implant integration and bone formation 3 months after implantation, whereas zoledronate showed the greatest potential. Regarding bone healing efficacy, the other two bisphosphonates, ibandronate and pamidronate, had minor impacts. Thus, a classification concerning the bone regenerative potential of the investigated bisphosphonates was similar to their release order [104]. Greiner and coworkers coated titanium implants with a synthetic PLA polymer. The coating included the nitrogen-containing bisphosphonate zoledronate for local drug release. The authors did not determine release kinetics in this study, but mentioned a potential drug elution of 50% within 2 days referring to a previous study. By investigating tibial fractures of rats for 84 days, zoledronate delivery resulted in increased mechanical stability compared to uncoated and unloaded controls [105]. In line with this, several investigators used coated titanium or tantalum implants for local delivery of zoledronate, alendronate, or pamidronate, respectively, to increase mechanical properties and bone ingrowth in vivo [106,107,108,109]. Furthermore, Li and coworkers used magnesium-based alloys and coated these implants with PLA-calcium phosphate composites incorporating zoledronate. In vitro, the authors detected a sustained drug release with a zoledronate release of 14% within the first 3 days based on diffusion and up to 27% in the following 3 weeks due to degradation of the implant coating. By investigating femoral fractures of osteoporotic rats for 12 weeks, local release of zoledronate increased bone repair and mechanical strength [110]. Thus, drug-eluting scaffold coatings of various components with several molecules such as growth factors and bisphosphonates, among others, displayed promising results in terms of bone regeneration and implant fixation.

#### 2.2.2. Hydrogels

Hydrogels are frequently studied with regard to potential applications in bone regeneration and fracture healing because of their injectability, degradability, tenability, and simple drug loading [36,111,112]. Growth factors or synthetic drugs can be incorporated into the hydrogel network covalently or non-covalently defining release kinetics in addition to hydrogel degradation-dependent drug delivery [113]. For instance, Fukui and coworkers conjugated simvastatin (250 µg/implant) into their gelatin hydrogel system with the intention to establish a slow-releasing drug delivery approach. In vivo, the locally applied drug carrier promoted femoral fracture healing in rats after 8 weeks [114]. Similarly, Yan and coworkers proved increased bone formation 4 weeks after application of a thermo-sensitive hydrogel with incorporated simvastatin in comparison to empty defects and unloaded scaffolds using a rat femoral defect model. In vitro, simvastatin showed a rapid release within the first 2 days. According to the authors, scaffolds released 80% of the incorporated simvastatin within 2 weeks demonstrating a correlation between drug delivery and hydrogel degradation [115]. By using photopolymerized hyaluronic acid-based hydrogels with encapsulated simvastatin, Bae and coworkers found accelerated bone healing of cranial defects in rabbits after 8 weeks. Regarding drug release kinetics, the authors described an initial burst release, which could be reduced by higher loading of the hydrogel carrier with simvastatin (up to 1 mg/scaffold) [116]. In addition, Tanabe and coworkers used photopolymerized gelatin hydrogels as drug carrier. The authors immersed the hydrogel disks in a fluvastatin (Figure 3) solution and indicated beneficial osteogenic effects in a rat calvarial defect model after 28 days. However, fluvastatin release kinetics were not studied [117]. Additionally, Chen and coworkers also followed up the approach of a gelatin hydrogel as drug delivery system. Their hydrogels contained 400 µg FGF-2 per 100 µL hydrogel and accelerated the bone healing in a rabbit tibial defect model in comparison to empty hydrogels after an investigation period of 8 weeks [118]. Besides animal studies, in a randomized, double-blind, placebo-controlled trial, Kawaguchi and coworkers reported an accelerated healing of fresh tibial fractures after injection of gelatin hydrogels incorporating FGF-2 [119]. Yamamoto and coworkers examined a gelatin-based hydrogel for controlled BMP-2 delivery. In vivo, BMP-2 release was dependent on the corresponding water content of the hydrogel, which modulated release kinetics from burst release within few days to sustained release over several weeks. A reduced water content resulted in a longer retention of BMP-2 within the hydrogel scaffold (99.7 wt%: complete elution within 15 days; 93.8 wt%: 60% remaining after 30 days). By investigating critical-size ulnar defect healing in rabbits, the authors showed an improved bone mineral density after administration of their BMP-2 (17 µg/scaffold) carrier system in comparison to BMP-2 without a carrier after 6 weeks [120,121]. Furthermore, Diab and coworkers incorporated BMP-2 (5 µg/200 µL scaffold) within a silk-based hydrogel. In vitro, the silk hydrogels released BMP-2 by burst release, whereas increasing silk concentration reduced release kinetics. In a rat critical-size femoral defect model, the authors implemented a nanofibrous PCL mesh in the bone defect area and subsequently filled with polymeric mesh containing their BMP-2-loaded hydrogel. The local growth factor delivery approach resulted in accelerated bone repair in comparison to unloaded silk scaffolds after 12 weeks [122]. In general, hydrogels can be useful drug delivery formulations with respect to their easy drug-loading properties and applicability, but hydrogel composition has to be carefully chosen to withstand the mechanical load in long bones. As shown in the last-mentioned study, other formulations like nanofibers for structural support can also be integrated into the scaffolds to build up a dual compound drug delivery carrier.

#### 2.2.3. Nanotubes and Nanofibers

Nanofiber- or nanotube-based scaffolds have been intensively studied in terms of bone repair due to their ECM-mimetic structure and morphology [36]. In general, drug delivery of nanotubes and nanofibers can be tuned by length or diameter of the tubes/fibers to delay drug diffusion and ensure controlled drug release profiles. Additionally, sequential drug release can be achieved by dual compound systems such as coated fibers or fibers with a surrounding polymeric scaffold [24,123]. Kwon and coworkers focused on nanotube implants consisting of titanium oxide soaked with zoledronate. By investigating a rabbit femoral model for 3 weeks, the authors found supported bone formation, but the release kinetics of the delivered bisphosphonate has to be determined yet [124]. In another approach, Shen and coworkers coated titanium oxide nanotubes with hydroxyapatite layers to efficiently load and release the anti-resorptive agent alendronate. In vitro, this drug delivery device enhanced osteoblast proliferation as alendronate exhibited a delayed release compared to nanotubes without hydroxyapatite layers. Beyond that, pH alterations varied drug release kinetics with an accelerated release at lower pH reflecting osteoclast microenvironment according to the authors. In vivo, the alendronate-releasing nanotube system improved bone formation in osteoporotic rabbits after 3 months [125]. Moreover, Lee and coworkers investigated the bone regenerative effect of BMP-2. The authors incorporated BMP-2 into heparin-binding amphiphilic nanofibers being embedded in a collagen scaffold. In vitro, BMP-2 displayed a delayed release in the presence of heparan sulfate as the scaffolds released only one third of loaded BMP-2 consistently after 8 days. In a critical-size femoral defect model being investigated over 6 weeks, the osteogenic effect of this nanofiber-based BMP-2 (1 µg/scaffold) dual compound delivery system was proven [126]. Further investigations on ectopic and cranial bone formation by sustained delivery of BMP-7 and simvastatin or BMP-2, respectively, demonstrated a good suitability of nanofibrous scaffolds in terms of bone regenerative applications [127,128,129,130]. Moreover, Kolambkar and coworkers established a dual compound drug delivery system, while the authors combined a nanofiber mesh forming a tube with an alginate hydrogel containing BMP-2 (5 µg/125 µL scaffold). BMP-2 delivery occurred almost completely within 7 days. In a rat critical-size femoral defect model, the described BMP-2 delivery approach enhanced bone repair after 12 weeks [131,132]. Regarding drug loading methods, Fu and coworkers investigated different loading techniques of PLGA-based fibrous scaffolds regarding drug release profiles and affected bone formation. In vitro, fibrous scaffolds with surface-absorbed BMP-2 showed burst release kinetics. In contrast, direct encapsulation of BMP-2 within the nanofibers during scaffold fabrication sustained BMP-2 release for several weeks. Moreover, the authors incorporated hydroxyapatite nanoparticles into the PLGA matrix. Furthermore, hydroxyapatite concentrations modulated BMP-2 release kinetics with higher hydroxyapatite content accelerating drug release. In a murine critical-size tibial defect model, the authors confirmed bone regenerative properties of their BMP-2 (1 ng/implant) drug delivery system 6 weeks after implantation [133]. Additionally, Zhu and coworkers investigated core-shell structured nanofibrous membranes for sustained rhBMP-2 delivery. The growth factor was incorporated in a PEG-based core surrounded by a PCL shell. In vitro, BMP-2 release followed a linear correlation of the investigation period of 4 weeks with a release rate of 500 pg per day. In a rabbit critical-size calvarial defect model, BMP-2 delivery by this nanofiber-based approach enhanced bone repair after 12 weeks in comparison to empty defects and unloaded scaffolds [134]. Boerckel and coworkers compared the bone formation after application of their BMP-2-loaded alginate hydrogel, being filled in the bone defect after covering the defect ends with a nanofiber mesh, with clinically used BMP-2-absorbed collagen sponges. In critical-size rat femoral defects, BMP-2 release of the nanofiber-alginate scaffold was slower than growth factor release from collagen sponges and resulted in enhanced bone formation and mechanical strength after 12 weeks [135]. The studies mentioned above underline that drug-loaded nanofibers are suitable for bone regenerative applications, especially if the nanofibers are embedded in a dual compound scaffold.

#### 2.2.4. Particles

Nanoparticles or microspheres were often incorporated into bone scaffolds to modulate mechanical scaffold properties and drug release kinetics based on their size [36]. In literature, several in vitro and in vivo studies on nanoparticles as drug carriers have been summarized [136,137]. For instance, Cao and coworkers investigated the bone regenerative potential of BMP-2-loaded chitosan-based nanoparticles in a critical-size radial defect model. The release kinetics follow an initial burst due to swelling properties, followed by a gradual release based on degradation. To reduce the initial burst release, these nanoparticles were incorporated in a gelatin hydrogel network. In rabbits, the BMP-2-loaded nanoparticles being incorporated into a hydrogel improved bone repair in comparison to hydrogel-encapsulated BMP-2 after 12 weeks [138]. Similarly, Zhou and coworkers focused on BMP-2 delivery systems. The authors loaded the surface of hydroxyapatite microspheres having a fibrous nanostructure with BMP-2 and determined sustained release kinetics over several days in vitro. Furthermore, this delivery approach promoted bone healing in rat femoral defects over an investigation period of 8 weeks [139]. Li and coworkers compared the bone forming properties after application of polyurethane scaffolds being directly loaded with BMP-2 and polymeric implants with incorporated PLGA microspheres encapsulating BMP-2 (2 µg/scaffold). Encapsulation of BMP-2 within the microspheres reduced the burst release depending on the size and composition of the microspheres, but the scaffolds incorporating BMP-2 directly showed the most promising effect on bone formation in rat femoral defects after 4 weeks. Therefore, the authors suggested the burst release of BMP-2 followed by a sustained release to be essential in bone healing processes [140]. Another method for establishing a drug delivery system was used by Rahman and coworkers. The authors loaded PLGA-PEG particles with BMP-2 (1 µg/scaffold) and sintered a solid scaffold afterwards. In this study, BMP-2 was released over 3 weeks in vitro reflecting sustained release kinetics. In a mouse calvarial defect model being investigated over 6 weeks, the mentioned BMP-2 delivery approach resulted in enhanced bone formation of more than 30% in comparison to unloaded scaffolds and empty defects [141]. Chung and coworkers investigated a drug delivery system consisting of a heparin-functionalized nanoparticle-fibrin gel with incorporated BMP-2 (4 µg/scaffold). A sustained BMP-2 release over at least 2 weeks was visible in vitro and resulted in enhanced rat calvarial critical-size defect healing after 4 weeks [142]. On the contrary, particulate drug delivery approaches can also not be beneficial for bone repair as shown in a study by Henslee and coworkers. The authors established a dual compound scaffold comprising PLGA microparticles with adsorbed BMP-2 and a surrounding solid, porous polymeric scaffold for structural support. The incorporation of BMP-2-loaded microspheres within a surrounding scaffold is supposed to reduce burst release and ensure sustained release kinetics. In a critical-size rat femoral defect model; however, this BMP-2 delivery system did not result in accelerated fracture healing over an investigation period of 12 weeks. The authors suggested that the solid scaffold could be, on the one hand, a physical barrier for bone forming cells hindering migration and gap closure and, on the other hand, a barrier for optimal BMP-2 delivery, whereby BMP-2 is needed especially in early bone healing phases and a delay might be disadvantageous [143]. Regarding growth factor delivery by particular carriers, Link and coworkers loaded microparticles comprising calcium phosphate and gelatin with TGF-ß (250 ng/scaffold). According to the authors, the TGF-ß delivery approach is supposed to have sustained release kinetics. However, no significant improvement of bone strength or bone formation was obvious 12 weeks after implantation in rabbit femoral defects comparing TGF-ß-loaded microparticles with empty composite carriers [144]. Lee and coworkers loaded collagen-chitosan microgranules with TGF-ß (100 ng/20 mg microgranules) and proved enhanced bone healing in a rabbit calvarial defect model after 4 weeks [145]. Several investigators including Patel and coworkers as well as Ennett and coworkers studied the release kinetics of VEGF from microparticles being incorporated in polymeric scaffolds in vitro and in vivo. Consequently, both crosslinking degree of the encapsulated microparticles and composition of the surrounding scaffold defines the release kinetics and modulates the initial burst [146,147]. This reflects that in a dual compound drug delivery system variation of each structural component influences release kinetics of the encapsulated drug decisively.

**Figure 6:**
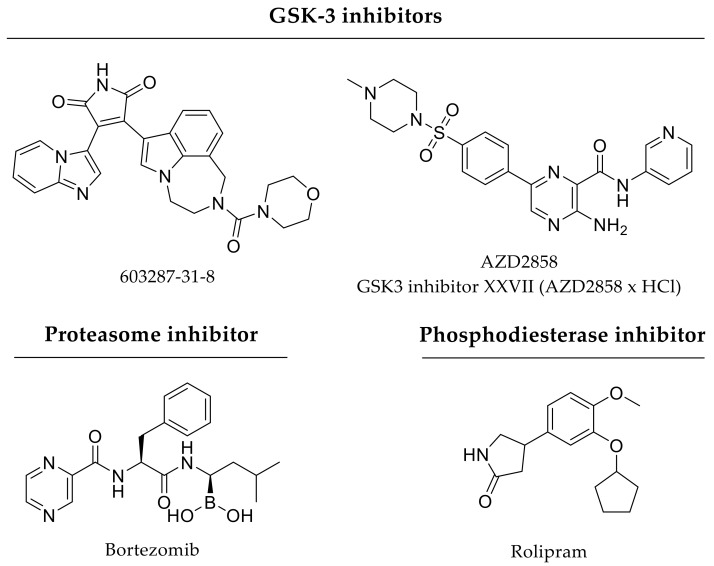
Chemical structure of selected enzyme inhibitors. GSK-3ß is a key enzyme of the Wnt/ß-catenin pathway and inhibition results in cytosolic accumulation of ß-catenin and further transcription of target genes promoting bone formation. Proteasome inhibitor bortezomib, mainly used in treatment of multiple myeloma, hinders proteasomal degradation of ß-catenin leading to the effects mentioned above. Furthermore, phosphodiesterase-4 inhibitors are anti-inflammatory agents and stimulate cellular proliferation as well as differentiation due to accumulated cGMP (cyclic guanosine monophosphate) and protein kinase G-mediated downstream signaling.

Khajuria and coworkers combined hydroxyapatite and zoledronate in a nanoparticulate delivery system to address bone formation and suppress bone resorption, respectively. Hydroxyapatite nanoparticles adsorbed zoledronate with a loading efficiency between 28% and 52%. In vitro, this drug delivery approach elicits burst release kinetics as over 60% of the incorporated drug was released after one hour. Nevertheless, zoledronate delivery improved bone properties compared to sole administration of hydroxyapatite or zoledronate in an osteoporotic rat model being investigated over 3 months [148]. Garrett and coworkers incorporated lovastatin in polymeric nanobeads. The authors showed a constant release of lovastatin with 25% cumulative release over 10 days in vitro. In vivo, this drug delivery approach improved bone healing and strength in a rat femoral model after 4 weeks [149]. In comparison, the lovastatin delivery system of Yoshii and coworkers exhibits faster release kinetics in vitro. Their drug delivery approach comprised lovastatin-loaded microparticles (2833 µg/cm^3^ defect) being incorporated into a polymeric scaffold and about 80% of lovastatin was released after 5 days. Furthermore, in a critical-size rat femoral bone defect model the drug delivery approach enhanced bone formation after 8 weeks [150]. Tai and coworkers investigated PLGA-hydroxyapatite microspheres loaded with simvastatin (3 or 5 mg/scaffold) in terms of bone healing within 4 weeks. Simvastatin showed a burst release at day one followed by a sustained delivery over 2 weeks in vitro. The authors suggested diffusion of the surface-adsorbed simvastatin to be responsible for the initial burst. In a murine gap fracture model, this drug delivery approach displayed bone regenerative potential [151]. Nyan and coworkers investigated tricalcium phosphate particles adsorbed with simvastatin (loading efficiency of 93.4 ± 5.8%). About 25% of the incorporated simvastatin was released within one day followed by a sustained release over 2 weeks. In vivo, this drug delivery system improved bone healing of rat calvarial defects after 21 days [152]. A hybrid drug delivery system was also in the focus of investigation by Lourenço and coworkers. The authors incorporated strontium-loaded hydroxyapatite microspheres within an alginate hydrogel also containing strontium. As strontium is present in both components of the drug delivery system, the authors supposed strontium to be released with different release kinetics over long periods. In vivo, strontium delivered by this approach over 60 days promoted bone formation in a critical-size femoral defect [153]. Das and coworkers investigated polymeric microspheres with incorporated FTY720. Within this study, different microsphere compositions reflecting fast-degrading and slow-degrading scaffolds were studied. The scaffold composition of the slow-degrading microspheres was more hydrophobic than the fast-degrading ones, with the slow-degrading microspheres having faster release kinetics in comparison to fast-degrading microspheres. The authors suggested a surface-near position of FTY720 within the hydrophobic slow-degrading microspheres to be responsible for the faster release. In a critical-size cranial defect model, FTY720-loaded microspheres enhanced bone formation over an investigation period of 9 weeks [154]. In a similar study, polymeric films were loaded with microspheres encapsulating the sphingosine 1-phosphate receptor agonists FTY720 or VPC01091 (Figure 5). These dual compound scaffolds were implanted for 6 weeks in critical-size rat cranial defects. In vivo, the drug delivery approach enhanced both bone formation and angiogenesis [155]. Wang and coworkers dealt with polymeric nanoparticles having a bone-targeting moiety for site-directed delivery of a GSK-3ß inhibitor XXVII (Figure 6). Inhibition of glycogen synthase kinase-3β (GSK-3β) activates Wnt/β-catenin signaling and, by this, regulates both osteoblast and osteoclast differentiation [156]. In terms of drug release, the drug delivery system showed higher GSK-3ß inhibitor release rates at lower pH compared to physiological pH over several days. In transgenic mice, these functionalized nanoparticles accumulated at the bone fracture and led to increased bone mineralization and formation after 4 weeks [157]. Additionally, other GSK-3 inhibitors like 603287-31-8 or AZD2858 could be promising adjuvant drugs, whereas their bone regenerative potentials have so far only been shown after oral administration or subcutaneous injection [22]. Particles as drug delivery systems provide a huge variety and tunability regarding composition, size, or functionalization with bone-targeting moieties and, by implication, drug loading, and release kinetics, too.

#### 2.2.5. Liposomes and Micelles

Liposomes and micelles as drug carriers can easily be designed to target bone defects specifically to increase drug efficacy and reduce undesired side effects. Therefore, liposomal or micellar surfaces are decorated with several bone-targeting moieties. For instance, bisphosphonates and peptides made out of negatively charged amino acids are able to bind to bone matrix component hydroxyapatite [18,23,158,159,160,161]. A bone-targeting drug delivery strategy by Xie and coworkers deals with PEG-based micelles shielding encapsulated atorvastatin (Figure 3). Drug release in this approach comprises an initial burst (up to 12 h) followed by decelerated release kinetic (up to 48 h). In osteoporotic rats, the authors depicted both an improved bone mineral density and mechanical strength resulting after a 12 week treatment with atorvastatin-loaded micelles [162]. For administration of water-insoluble drugs like simvastatin, Tanigo and coworkers developed a drug delivery system combining statin micelles together with a gelatin hydrogel. Simvastatin release proceeded in a sustained manner over 2 weeks due to dependency of drug delivery on unspecific gelatin degradation by collagenases. In vivo, the released simvastatin led to an accelerated bone formation in a rabbit mandibular model after 5 weeks [163]. Furthermore, Low and coworkers used an aspartic acid octapeptide as bone-targeting moiety and delivered a GSK3β inhibitor by micelles within 2 days. Treatment of femoral fractures with this drug delivery approach resulted in increased bone mineral density and bone volume due to upregulated β-catenin level [164,165]. In contrast, liposomal drug delivery can also be detrimental on bone healing as shown by Lin and O’Connor. The authors detected impaired fracture healing of femoral defects after treatment with clodronate-encapsulated liposomes (Figure 2) over an investigation period of 28 days [166]. Liposomes or micelles are suitable site-directed drug carriers, particularly for water-insoluble drugs due to surface-decorated bone-targeting moieties.

Besides positive effects on bone fracture healing through treatment with a bioactive, adjuvant drug, the major limitation of single drug delivery approaches is targeting a single physiological pathway in the complex process of bone healing. To improve bone repair and drug efficacy, drug delivery systems combining the action of several drugs of diverse substance types and classes in an additive or synergistic way are required. In this way, several cellular or molecular processes can be addressed at different healing phases by one drug delivery system.

## 3. Dual Drug Delivery

**Figure 7:**
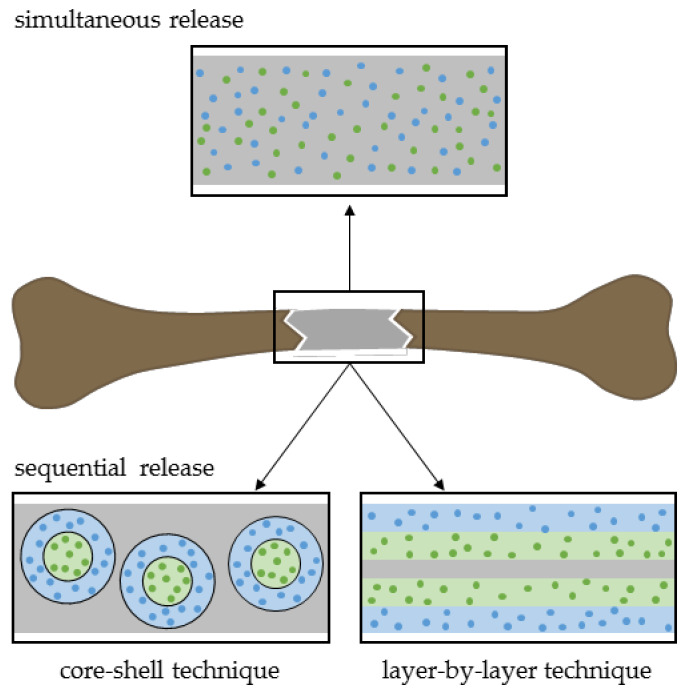
Exemplary drug loading techniques for simultaneous or sequential drug co-delivery.

Most commonly, therapy strategies have focused on drug release of a single compound. However, bone healing, as a complex process, requires a well-orchestrated interplay of multiple factors at certain bone repair phases to affect key events like osteogenesis, angiogenesis, and inflammation. By applying several drugs simultaneously or sequentially, potential synergistic effects might strengthen bone regeneration. For instance, combining angiogenic and osteogenic signals or anabolic and anti-resorptive effects are desirable. Kim and Tabata summarized a multitude of dual drug delivery systems for simultaneous or sequential drug release in vitro and in vivo [167]. The authors concluded that carrier composition for drug encapsulation, drug ratio as well as time and place have to be considered to establish an optimal delivery system. Thereby, dual drug loading can rely, for instance, on layer-by-layer or core-shell strategies (Figure 7) [12,167,168]. In the following part, several dual drug delivery studies will be described in detail.

### 3.1. Growth Factors

In the literature, most investigations deal with growth factor co-delivery. Van der Stok and coworkers filled porous titanium scaffolds with gelatin gels containing BMP-2 (3 µg total dose) and FGF-2 (0.6 µg total dose). The scaffolds were supposed to release FGF-2 rapidly, whereas BMP-2 delivery was sustained. In rat femoral bone defects, scaffolds loaded with both growth factors enhanced bone formation compared to unloaded ones after 12 weeks. However, the dual delivery approach did not significantly improve bone regeneration in comparison to single growth factor delivery [169]. Wang and coworkers made use of FGF-2 and BMP-2 as well. The authors loaded the core and shell of their microspheres with both growth factors for designed sequential release. The most promising results in bridging autologous bone grafts in tibial defects were obviously for the delivery system with BMP-2 in the core and FGF-2 in the shell region. Thereby, FGF-2 was released first within 2 weeks followed by sustained BMP-2 delivery over 4 weeks [170].

Regarding dual delivery of angiogenic and osteogenic molecules, Hernández and coworkers used polymeric microspheres with incorporated VEGF and BMP-2 within a porous scaffold. In vivo, delivery of both growth factors showed a gentle burst followed by a sustained release proceeding over 3 to 4 weeks. The authors found only short-term synergistic effects of their dual delivery system by investigating femoral defects in rabbits. For a more beneficial bone regenerative application, further optimizations of the co-delivery strategy such as drug ratio have to be considered [171]. In another strategy to create sequential release profiles, Geuze and coworkers loaded calcium phosphate scaffolds with microparticles and surrounding gelatin hydrogel determining sustained or fast release, respectively. Both delivery materials contained either BMP-2 or VEGF. The authors analyzed the impact of different release profiles on bone formation. In an ectopic and critical-size ulnar defect model followed up for 9 weeks, dual growth factor delivery was beneficial. In contrast to ectopic bone formation, where BMP-2 timing had an effect, in the critical-size defect no difference in formed bone amount for fast or slow delivery of both growth factors was obvious [172]. Exploiting the same growth factors and analogous delivery strategy, Kempen and coworkers fabricated scaffolds containing BMP-2-loaded microspheres and surrounding VEGF-loaded gelatin hydrogel. The drug carrier released both growth factors sequentially in vivo. VEGF release showed an initial burst within 3 days followed by sustained BMP-2 delivery over several weeks. The authors found an improved bone regeneration in case of ectopic application of this dual delivery approach compared to single treatment, but no significant improvement in an orthotopic femoral defect model after 8 weeks [173]. In contrast, Sukul and coworkers established a calcium phosphate-based scaffold loaded with nanocellulose. Within the nanocellulose, VEGF and BMP-2 were incorporated for dual sustained release. In vivo, this dual drug delivery system enhanced bone formation in an orthotopic model, whereas no desired effects were obvious in an ectopic model after 4 weeks [174]. Moreover, by analyzing ectopic bone formation, Shah and coworkers found promising effects of co-delivered BMP-2 and VEGF from multilayered films. In vitro, rhVEGF being part of the top layer was eluted within 7 days, whereas rhBMP-2 release from the lower layer of the fabricated scaffold lasts for 2 weeks. In comparison to single BMP-2 release, co-delivery increased bone mineral density by almost a third after 9 weeks in vivo [175]. Additionally, a variety of in vivo investigations regarding co-delivery systems analyzed bone regeneration in cranial defects. Studies include dual delivery of angiogenic VEGF and osteogenic BMP-2 [176,177,178]. For instance, Subbiah and coworkers investigated dual compound scaffolds comprising PLGA nanoparticles with encapsulated BMP-2 (80% loading efficiency) for fast release and surrounding alginate microcapsules with incorporated VEGF (40% loading efficiency) for sustained release. In rat cranial defect, this dual drug delivery system showed synergistic effects and accelerated bone regeneration after 56 days in comparison to single BMP-2 treatment [178].

Moreover, some investigators have used multilayered coated membranes incorporating BMP-2 in bottom layers and PDGF in top layers for promoted bone repair over 4 weeks in a calvarial defect model. Both growth factors exhibited sustained release kinetics in vitro and in vivo, whereas PDGF release in vivo was faster in comparison to BMP-2 as degradation of the coatings proceeds from top to bottom [179]. Oest and coworkers also investigated dual growth factor delivery approaches. The authors used polymeric scaffolds with RGD-alginate hydrogels. The hydrogels itself incorporated BMP-2 (200 ng/50 µL scaffold) and TGF-ß (20 ng/50 µL scaffold). Co-delivery of the mentioned growth factors resulted in increased bone volume of rat femoral defects after an investigation period of 16 weeks, but did not led to sufficient bone union [180]. Moreover, TGF-ß release combined with IGF (insulin-like growth factor) seems promising. In rats, implantation of IGF- and TGF-ß-coated titanium implants enhanced biomechanical stability based on dual growth factor delivery. Especially during early bone healing phases, growth factor delivery from polymeric coating was beneficial. This was due to a rapid release of half of the loaded growth factors within the first 2 days [181,182,183].

Additionally, co-delivery of BMP-2 with SDF-1 (stromal derived factor-1), a chemokine, could arouse synergistic bone regenerative potential, as shown by Ratanavaraporn and coworkers. Their chemically crosslinked gelatin-based hydrogel system released the incorporated molecules rapidly during the first days after implantation due to diffusion mechanisms, followed by a degradation-dependent delivery over 3 weeks. Comparing release of BMP-2 (300 ng/scaffold) with SDF-1 (500 ng/scaffold), the authors observed a faster SDF-1 release in the presence of BMP-2 and suggested a stronger interaction of the gelatin hydrogel with BMP-2 compared to SDF-1. In vivo, the dual delivery improved bone regeneration of an ulnar critical-size defect in rats after 4 weeks in comparison to single BMP-2 treatment [184]. Sequential delivery of BMP-2 and SDF-1 was also subject of investigation in a study by Shen and coworkers. The authors fabricated scaffolds with two compounds including BMP-2-loaded microspheres and a hydroxyapatite-based scaffold functionalized with adsorbed SDF-1. SDF-1 release followed a characteristically initial burst during the first days, whereas BMP-2 delivery proceeded in a sustained manner over 3 weeks. In vivo, synergistic effects of both delivered molecules enhanced bone repair in rat cranial defects 12 weeks after implantation [185]. Zwingenberger and coworkers found a synergistic effect of SDF-1α (10 µg/scaffold) and low dose BMP-2 (2.5 µg/scaffold) on bone regeneration in a murine critical-size bone defect model by co-release from heparinized mineralized collagen type I matrix scaffolds over 6 weeks [186]. Thus, dual delivery of different growth factors to stimulate osteogenesis and angiogenesis appears to be a promising approach.

### 3.2. Growth Factors and Bisphosphonates

In addition to dual delivery of angiogenic and osteogenic factors, co-delivery of the most promising osteogenic growth factor BMP with bisphosphonates addresses several cell types being important during bone regeneration. To combine anabolic and anti-resorptive effects, Murphy and coworkers incorporated rhBMP-2 (5–10 µg/scaffold) and zoledronate (2–10 µg/scaffold) into a collagen–hydroxyapatite scaffold. In vitro, BMP-2 release displayed a characteristic initial burst. In contrast, the authors suggested a longer retention of zoledronate within the scaffold as the bisphosphonate has a high binding affinity to the hydroxyapatite compound. Within this approach, the authors demonstrated an almost 6-fold increased ectopic bone formation by dual delivery of growth factor and bisphosphonate in comparison to single BMP-2 treatment [187]. Similarly, Yu and coworkers analyzed the effect of a drug-loaded polymeric scaffold on bone healing up to 8 weeks. In their studies, the scaffolds contained either BMP-7 and pamidronate or BMP-2 and zoledronate. According to the authors, PLGA scaffolds delayed drug delivery in comparison to clinically used collagen scaffolds. Addressing both anabolic and anti-resorptive targets resulted in an enhanced bone formation [188,189]. Gao and coworkers loaded hydroxyapatite-coated titanium implants with zoledronate (1 mg/mL) and basic FGF (bFGF, 20 µg/mL) to stimulate bone formation and suppress bone resorption simultaneously. In vitro, scaffolds with single drug coating exhibited burst release kinetics especially during the first 3 days. In comparison, initial burst release of the adsorbed bisphosphonate and growth factor from scaffolds with both drugs was reduced. In osteoporotic rats, combined local administration of zoledronate and bFGF by this coating approach resulted in accelerated bone formation and mechanical strength compared to single drug coatings after 3 months [190].

### 3.3. Growth Factors and Enzyme Inhibitors or Receptor Agonists

**Figure 8:**
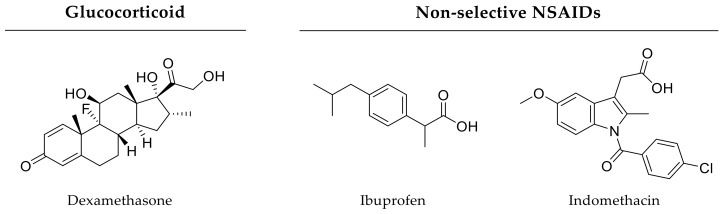
Chemical structures of selected anti-inflammatory drugs. Both glucocorticoids and NSAIDs are inflammation-modulatory small molecules inhibiting cyclooxygenase isoforms and prostaglandin production. These drugs mainly impair bone repair, as prostaglandins are crucial during early bone healing phases.

In further ectopic bone formation experiments, Tokuhara and coworkers, as well as Toyoda and coworkers, used PEG implants for drug delivery and proved beneficial effects regarding combined release of rhBMP-2 and rolipram (phosphodiesterase-4 inhibitor, Figure 6) or a prostaglandin receptor agonist, respectively [191,192]. Furthermore, simultaneous delivery of a prostaglandin E2 receptor agonist and BMP-2 being incorporated in a hydrogel accelerated bone formation in murine calvarial defects [193]. In contrast, Li and coworkers developed a drug delivery scaffold for sequential delivery of the glucocorticoid dexamethasone (Figure 8) and BMP-2. Thereby, BMP-2 was encapsulated in nanoparticles and a surrounding polymer contained dexamethasone resulting in different release kinetics. More precisely, dexamethasone delivery proceeded over 7 days, whereas BMP-2 release lasted for 4 weeks. In vivo, delivery of osteogenic BMP-2 and anti-inflammatory (antiphlogistic) glucocorticoid resulted in enhanced bone repair of rat calvarial defects compared to single treatment approaches over an investigation period of 12 weeks [194]. Furthermore, delivery of small molecule drugs and chemokines like SDF-1 are in focus of bone healing research. In vivo, co-delivery of simvastatin with SDF-1 from PLGA scaffolds promoted bone formation in murine critical-size calvarial defects after 6 weeks in comparison to single drug treatments [195,196]. Additionally, Kim and coworkers investigated bone formation in rat critical-size bone defects after application of gelatin hydrogels incorporating SEW2871-loaded micelles and platelet-rich plasma (PRP). Both molecules were released in vivo within 7 days due to gelatin degradation. In terms of bone repair, sphingosine 1-phosphate receptor agonist SEW2871 (Figure 5) alone was not sufficient, but the dual delivery approach resulted in accelerated bone healing 6 weeks after implantation [197].

### 3.4. Growth Factors and Antibiotics

**Figure 9:**
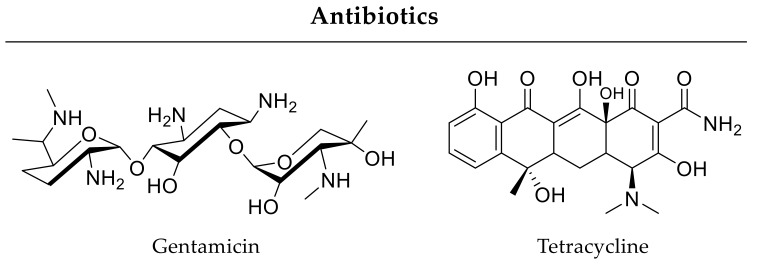
Chemical structures of selected antibiotics. Gentamicin prevents bone infections, whereas tetracycline inhibits osteoclast differentiation and displays a high affinity to bone minerals as well.

In addition to improving bone healing in principle, therapy approaches should also combat adverse infections hindering appropriate regeneration of critical-size bone defects. For example, Min and coworkers designed implant coatings for combined delivery of antibiotics and osteoinductive factors. Within this strategy, coatings of an orthopedic implant contained BMP-2 in the lower layer for sustained release and the aminoglycoside antibiotic gentamicin (Figure 9) as the top layer for burst release. In a rat tibia model studied for 8 weeks, the authors proved the antibiotic and osteoinductive effects of their dual delivery system [198]. Furthermore, combinations of antibiotic agents with other small molecule drugs are possible. For instance, calcium phosphate-based carrier released antibiotic tetracycline (Figure 9) together with anti-inflammatory ibuprofen (Figure 8) by burst release kinetics resulting in accelerated bone formation in rat cranial defects after 12 weeks compared to controls without a drug carrier [199].

### 3.5. Growth Factors and Cells

Besides co-delivery of several molecules, the research focus of bone healing strategies is also on cell-based therapy approaches. Decambron and coworkers analyzed the bone regenerative effects of mesenchymal stem cells and BMP-2 being co-delivered from calcium-based scaffolds. The authors adsorbed BMP-2 in low dose onto the scaffold, but did not determine the protein release kinetics. In vivo investigations focused on bone repair using a sheep model. This co-delivery approach tended to result in accelerated bone formation in comparison to single cell or BMP-2 application, respectively. However, only half of the bone defects were healed, demonstrating the necessity of further improving the drug delivery strategy [200]. On the contrary, the study by Kirker-Head and coworkers showed enhanced bone regeneration of rat critical-size femoral defects after application of BMP-2 and human mesenchymal stem cells loaded on silk scaffolds as well as cell-free scaffolds [201]. Some investigations went a step further towards co-delivery of cells and several growth factors to address diverse therapeutic targets. Thereby, Kanczler and coworkers seeded bone marrow stromal cells (BMSCs) on scaffolds comprising osteogenic BMP-2 and angiogenic VEGF. Concerning sequential drug release, the authors fabricated a dual compound scaffold. In terms of scaffold composition, VEGF was encapsulated within alginate fibers for fast release kinetics, which in turn were embedded in polymeric BMP-2 carrier for decelerated delivery of the second growth factor. In a critical-size femoral defect model, the authors showed enhanced bone formation upon dual delivery approach of BMSCs and both growth factors in comparison to empty scaffold and cell-free scaffold [202]. Simmons and coworkers also followed dual growth factor release combined with cell delivery in their investigations. The scaffolds contained additional rhBMP-2 and rhTGF-ß3 without designed release kinetics. The authors found accelerated bone healing regarding dual delivery of growth factors in comparison to single treatment approaches after application of the BMSC-containing alginate hydrogels ectopically to mice [203]. Furthermore, mixtures of multiple physiologically occurring growth factors derived from adipose tissue, PRP and conditioned medium from hypoxia-treated human telomerase immortalized BMSCs have a high bone-regenerative potential in first in vitro investigations [204]. These mixtures may lead to a synergism between chemoattractive potential and osteogenic and angiogenic differentiation capacity of the angiogenic proteins and cytokines.

In general, dual delivery approaches seem to be very promising due to targeting several key aspects in bone healing. Further optimizations of these systems have to be considered with regard to drug ratio, release kinetics or spatiotemporal delivery patterns.

## 4. Triggered Drug Delivery

**Figure 10:**
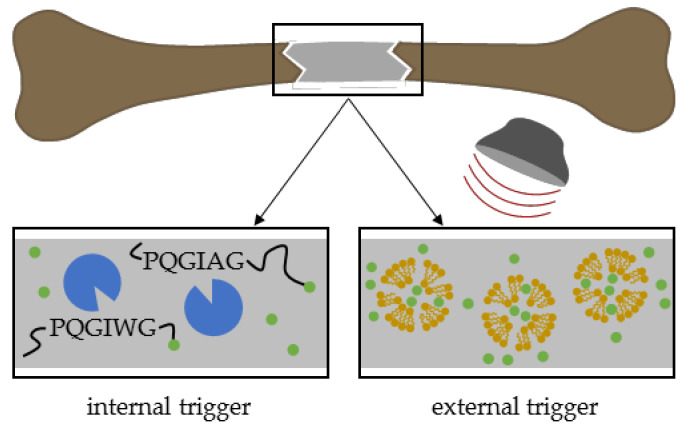
Exemplary targeted drug delivery approaches based on internal or external stimuli.

During crucial bone healing phases, controlled drug release as a response to a certain stimulus at a defined time point represents an improvement of current therapy strategies. Triggered drug delivery reduces the initial burst release, which is typical for diffusion-dependent drug release. On-demand release kinetics optimize drug specificity, quantity, and reduce drug toxicity. The physiological environment including active enzymes and pH, or external stimuli like temperature, physical fields, light, or ultrasound (Figure 10), are feasible triggers in designed drug delivery systems [12,35,205,206,207,208,209,210,211,212,213,214,215].

### 4.1. Proteolytic Enzymes

The introduction of specific peptide sequences as enzymatically cleavable linkers into scaffold compounds enables protease-mediated or site-directed degradation-dependent drug release. For instance, matrix metalloproteases (MMPs) are key enzymes involved in several bone healing phases and MMP cleavage sites are interesting features for designing smart drug carriers. Garcia and coworkers used hydrogels containing a VPM-sequence (VPMS↓MRGG) for protease-dependent drug delivery. The authors studied VEGF-loaded PEG-based hydrogels having additional integrin-specific peptide sequences. VEGF was covalently bound to the hydrogel network to ensure degradation-dependent release kinetics. In vitro, treatment with collagenase eluted VEGF within 2 days, but the authors supposed longer drug release properties in vivo. In a murine critical-size radial bone defect model, controlled VEGF release increased vascularization and bone formation dependent on the integrated integrin structure after 8 weeks [216]. In rat calvarial defects, Kim and coworkers showed enhanced bone regeneration after application of hyaluronic acid-based hydrogel. These hydrogels were MMP-sensitive, as they comprised an MMP cleavage site (GCRDGPQG↓IWGQDRCG). Furthermore, the hydrogel network contained BMP-2 (800 ng/construct) for local drug release and stem cells (8 × 10^5^ cells/construct). Application of this drug delivery system improved bone formation based on an assumed synergistic interplay of BMP-2 and stem cells after 4 weeks [217]. Lutolf and coworkers incorporated the same MMP-cleavable sequence in a PEG-based hydrogel. In their study, the authors showed an MMP-2-triggered BMP-2 (5 µg/defect) release and accelerated bone healing of rat critical-size cranial defects 5 weeks after implantation [218]. Similar findings were also shown in a study by Terella and coworkers, whereas another cleavable MMP linker sequence (KKCGGPQG↓IAGQGCKK) was used [219].

In addition to MMP-sensitive sequences, several studies investigated enzymatically triggered drug release based on the introduction of other protease-cleavable linker. For instance, Hsu and coworkers focused on PEG-based hydrogels including a cathepsin K-degradable linker (GGGMGPS↓GPWGGK). The protease is highly expressed in osteoclasts shifting hydrogel degradation and degradation-dependent drug release towards later bone-related processes like bone remodeling. By using cathepsin K in vitro, the authors showed rapid degradation and release kinetics within 24 h. In contrast, collagenase treatment delayed degradation indicating a cathepsin K-selective drug release. Furthermore, cell culture experiments proved the osteoclast-specific response towards this hydrogel system. Therein, the hydrogel was degradation-resistant against osteoblasts, but osteoclasts were feasible to degrade it [220]. Pan and coworkers studied another cathepsin K-sensitive peptide linker (Gly Gly Pro-Nle-alcohol moiety; GGPJ↓-alcohol moiety) within a bone-targeting, acrylamide-based polymer for prostaglandin E1 (PGE1) delivery. PGE1, a physiologically active eicosanoid having diverse hormone-like effects, is used as an anabolic drug to support bone formation and is only biologically active, when being released from the polymer in this delivery system. Thereby, PGE1 release kinetics depend on the PGE1-containing side chains according to the cathepsin K assay [221]. The authors also investigated the effect of their drug delivery system on bone formation in an ovariectomized rat model. The release of bioactive PGE1 resulted in higher bone mineral densities after 4 weeks [222]. In another approach, delivery of simvastatin from self-assembled PEG-based micelles was in focus. In vivo, the micelles are supposed to target the bone defect site and release conjugated simvastatin due to hydrolysis of ester bond linkages. The authors showed a gradual release of simvastatin and concomitant promotion of bone healing in femoral fractures after 21 days [223,224]. Taken together, the introduction of enzymatically cleavable linker within the scaffolds are promising strategies for targeted drug delivery with several proteases and linker sequences being available.

### 4.2. Redox Environment

A redox-triggered drug delivery approach based on the cellular microenvironment could be another interesting stimulus for controlled drug release. Yang and coworkers examined the impact of disulfide-containing PEG-based scaffolds with incorporated rhBMP-2 (50 µg/scaffold) on bone healing. In this drug delivery approach, oxidative stress produces glutathione, which triggers scaffold degradation. The degradation profile was dependent on redox and polymer concentration, resulting in a tunable degradation from several minutes up to several weeks. To simulate drug release in vitro, the authors used bovine serum albumin as model compound and detected an initial burst release within 4 days, with higher polymer concentrations retarding the release kinetics up to 3 weeks. In vivo, the impact of the sustained rhBMP-2 release resulted in an improved bone repair and union of rabbit radial critical defects after 12 weeks [225]. Likewise, Gong and coworkers took advantage of the glutathione-mediated drug delivery mechanism. The authors investigated redox-sensitive nanofibers for BMP-2 delivery. As with the former investigation, in vitro BMP-2 release followed burst release kinetics and accelerated bone regeneration in a rat mandibular defect model after 12 weeks due to redox-triggered BMP-2 release was confirmed [226]. However, only a few investigations have focused on redox-sensitive drug delivery approaches in terms of bone healing so far.

### 4.3. pH Alteration

To remain in the field of microenvironmental stimuli, site-directed drug delivery based on pH alterations at a cellular level represents an attractive research approach. Within a complex and challenging drug delivery strategy, Gan and coworkers developed a pH-responsive drug delivery system based on chitosan-functionalized mesoporous silica nanoparticles. The system serves for dual release of BMP-2 (15 µg/scaffold) and dexamethasone (20 µg/scaffold), which were non-covalently bound to the chitosan coating or encapsulated within the nanoparticles, respectively. Dexamethasone is a glucocorticoid and operates as anti-inflammatory drug, but also might evoke detrimental effects of steroid administration on bone regeneration. In a physiological context, the chitosan-coated nanoparticles are supposed to elute BMP-2 quickly in the cytosol, whereas encapsulated dexamethasone is intended to be released in a controlled manner after efficient endocytosis into cells and at a more acidic pH value within lysosomes. The dual delivery strategy addresses optimal accessibility of membrane-bound BMP-2 receptors and intracellular glucocorticoid receptors for activating different signaling pathways synergistically. Moreover, this delivery approach might prevent time- and concentration-dependent adverse effects of dexamethasone treatment. The authors found an initial burst release within 12 h for the surface immobilized BMP-2 and pH-dependent release kinetics for dexamethasone. The nanoparticles eluted dexamethasone within 1 h, when bringing the system in a slight acidic pH around 6.0, but no drug was released in neutral pH. Therefore, pH-dependent opening and closing of the mesopores for several cycles switched drug release. Besides stimulating osteoblast differentiation in vitro, the application of the described drug delivery approach also enhanced ectopic bone formation in vivo after 4 weeks [227].

### 4.4. Temperature

In addition to internal triggers, external stimuli, like temperature, are also in the focus of research for targeted drug delivery in terms of bone regeneration. López-Noriega and coworkers described an approach using drug-containing thermo-sensitive liposomes being covalently linked to an osteoconductive collagen-hydroxyapatite scaffold. A thermal pulse of 42 °C stimulated on-demand release. The stimulus introduced a phase transition of the liposome membrane and, in consequence of membrane permeabilization, an altered drug release up to 5-fold increase was achieved. In vitro, a third of loaded osteogenic protein PTHrP (parathyroid hormone-related protein) was released rapidly reflecting the amount of surface-adsorbed peptide. According to the authors, a slow PTHrP release over 14 days followed burst release due to peptide leakage from liposomes under standard cell culture conditions. Upon this thermo-responsive drug delivery approach, PTHrP was still bioactive [228]. In a second thermo-responsive approach, Reis and coworkers reported a controlled drug delivery from nanoparticle-based coatings. The authors incorporated bortezomib (Figure 6) as model drug in their coating and analyzed the release kinetics by in vitro investigations. Bortezomib is a proteasome inhibitor and a potential drug for therapy of bone fractures in the context of bone-affecting neoplasias such as multiple myeloma, as the drug can block inhibitors of the Wnt signaling, a crucial pathway in promoting bone formation. Similar to the first investigation, the authors found an initial burst release of adsorbed bortezomib followed by a gradual elution over 2 days. With a thermal stimulus of 42 °C, bortezomib release was accelerated due to conformational changes of the thermo-responsive coating [229]. Regarding thermally triggered drug release for bone regeneration, in vivo investigations are still lacking.

### 4.5. Near-Infrared Light Irradiation

As another external stimulus, light application can tune drug release kinetics of a respective drug carrier. In principle, near-infrared light absorbed by appropriate materials such as black phosphorus and following light-to-heat conversion is probably suitable for triggered drug delivery systems. For example, Wang and coworkers investigated a light-triggered strontium release based on a PLGA scaffold containing black phosphorus and strontium chloride in terms of bone regeneration. Strontium promotes osteoblast differentiation, bone fracture healing, and improves mechanical strength as well as inhibits osteoclast-dependent bone resorption. Upon this approach, strontium showed an initial burst release and subsequent elution within 4 weeks. By applying this drug delivery strategy in vivo, the authors proved an accelerated bone regeneration of rat femoral defects after 8 weeks [230].

### 4.6. Physical Fields

Other external stimuli including magnetic and electric fields are in the focus of scientific research regarding the development of controlled drug delivery strategies. With respect to magnetic field-induced drug release, Aw and coworkers developed titanium nanotube arrays including polymer micelles as drug delivery systems. In their methodological study, the authors showed a sequential release of a hydrophobic drug (indomethacin, Figure 8) and a hydrophilic drug (gentamicin) hereinafter. The nanotube array released indomethacin, a potent antiphlogistic drug, and antibiotic gentamicin independently over 10 days. The release kinetics for each individual release duration of 5 days displayed a burst release followed by residual diffusion [231,232]. Using magnetically triggered drug release, Matsuo and coworkers investigated injectable magnetic liposomes to deliver BMP-2 (3 µg total dose). To treat bone defects in a rat model over 9 weeks, the authors implanted a magnet and applied BMP-2 loaded liposomes topically. In vivo, the targeted BMP-2 delivery approach, being applied in early bone healing phases, enhanced bone formation in comparison to topical BMP-2 administration [233]. In addition to magnetic stimuli, studies report on electrically responsive drug delivery systems. Microreservoires made out of a conductive polymer deliver dexamethasone in response to an electric trigger. For electrically stimulated drug release, the authors applied voltage cycles between −1 V and +1 V. Besides a passive drug diffusion of small dexamethasone amounts starting after 50 h, the electric stimuli triggered an abrupt release. The on-demand release of dexamethasone resulted in enhanced osteogenic differentiation in in vitro experiments [208].

### 4.7. Ultrasound

For tunable on-demand drug delivery, ultrasound-triggered systems represent another possible tool. Crasto and coworkers used sono-disruptable liposomes for controlled BMP-2 delivery. In a proof-of-principle study, the authors trapped BMP-2 within liposomes and BMP-2 release as a response to ultrasound exposure followed fast release kinetics. Moreover, the drug delivery system enhanced ectopic bone formation 28 days after implantation in a murine model [234].

Triggered delivery systems (Table 2) are advantageous with respect to time-specific drug release to address critical bone healing processes precisely. In particular, investigations of external triggered delivery approaches remain in experimental setups so far and evidence in substantial in vivo studies are lacking [34]. Nevertheless, protease-mediated drug delivery approaches already show promising results.

## 5. Conclusions

Numerous targeted drug delivery approaches have been employed to enhance bone healing. Smart scaffolds should provide structural and biological features according to the ‘diamond concept’ to reduce adverse side effects and increase local drug efficacy. For designing drug delivery implants, a variety of natural and synthetic polymers, or hybrid compounds, that showed promising results in vivo are available. These drug carriers can be fabricated in different formulations such as drug-eluting implant coatings, hydrogels, particles, fibers, or dual compound systems. Moreover, bone-targeting properties or other bioinspired and bioattractive surface functionalizations can be included. Regarding drug delivery in the crucial bone healing phases, drug concentration and release kinetics have to be taken into consideration when loading the drug carrier [20,21,22,235]. Thereby, several drug-loading techniques like core-shell or layer-by-layer loading can be applied to modulate release kinetics of single drugs from burst to sustained release or even co-deliver drugs simultaneously or sequentially.

In terms of clinical translation of local drug delivery systems for bone regenerative applications, drug-loaded scaffolds should optimally be fabricated hierarchically for spatiotemporal drug release of several drugs. A particular challenge is the time-controlled release of a combination of highly hydrophobic and highly hydrophilic drugs. Innovative approaches would, for example, enable the targeted use of selective cyclooxygenase-2 inhibitors, which are highly potent anti-inflammatory drugs [20,236,237]. To achieve controlled release profiles, the smart scaffolds are supposed to have multifactorial properties such as stimuli-triggered drug release characteristics and surface modifications for optimal cell adhesion, and nutrient supply [123,238].

## Figures and Tables

**Table 1:** Summary of selected adjuvant drugs for local drug delivery approaches being discussed in this work and their effects on bone fracture healing. Their molecular targets, mechanisms of action, direct effects on bone metabolism and side effects have been described in detail elsewhere [20,21,22].

Adjuvant Drugs	Effect on Bone Metabolism
***Growth factors***	act during all fracture healing stages; stimulate proliferation and differentiation of bone forming cells as well as angiogenesis
BMP-2/BMP-7	
FGF-2	
IGF	
PDGF	
TGF-ß	
VEGF	
***Hormones***	acts during all fracture healing stages; anabolic and catabolic effects on bone healing depending on dose and administration
Parathyroid hormone	
***Bisphosphonates***	act during several fracture healing stages; prevent bone resorption and increase bone mineralization
*Nitrogen-containing bisphosphonates*	
Alendronate	
Ibandronate	
Pamidronate	
Zoledronate	
*Non-nitrogen-containing bisphosphonates*	
Clodronate	
***Glucocorticoids***	interferes in late fracture healing phases; inhibits osteoclasto- and osteoblastogenesis; low dose of short-acting glucocorticoids may not be adverse
Dexamethasone	
***Non-steroidal anti-inflammatory drugs (NSAIDs)***	elicit anti-inflammatory effects due to inhibition of cyclooxygenases and reduction of prostaglandin production; mainly impair bone repair, especially during the first crucial bone healing phases
Ibuprofen	
Indomethacin	
***Prostaglandins***	important during early fracture healing phases; biphasic effect on osteoblasts and osteoclasts; intermittent application recommended
Prostaglandin E1	
Prostaglandin receptor agonist	
***Enzyme inhibitors***	
*GSK-3ß inhibitors*	GSK-3ß inhibitors prevent proteasomal degradation of β-catenin leading to cytosolic accumulation and nuclear translocation of β-catenin for transcriptional activation of various target genes
603287-31-8	
AZD2858 or	
GSK-3ß inhibitor XXVII (AZD2858 × HCl)	
*Phosphodiesterase-4 inhibitor*	phosphodiesterase-4 inhibitor elicits anti-inflammatory effects and increases proliferation and differentiation of osteoblasts and osteoclasts; low doses used for short-term treatment are recommended
Rolipram	
*Proteasome inhibitor*	proteasome inhibitor promotes osteoblastogenesis as well as inhibits osteoclastogenesis; low doses used for short-term treatment are recommended
Bortezomib	
***Sphingosine 1-phosphate receptor agonists***	increase angiogenesis and osteogenesis
FTY720	
SEW2871	
VPC0191	
***HMG-CoA (3-hydroxy-3-methylglutaryl-CoA) reductase inhibitors-statins***	promote osteogenesis and appear to be anti-inflammatory and pro-angiogenic
Lovastatin	
Pravastatin	
Simvastatin	
Atorvastatin	
Fluvastatin	
Pitavastatin	
Rosuvastatin	
***Divalent metal ions***	enhances bone formation and mechanical strength; suppresses bone resorption
Strontium	
***Antibiotics***	prevent bone infections; tetracycline inhibits osteoclast differentiation and is high affine to bone minerals
Gentamicin	
Tetracycline	

**Table 2:** Overview of representative studies providing kinetic data obtained from use of single or dual compound delivery systems and passive or triggered drug release.

Drug Delivery System	Single (S) or Dual (D) Compound	Drug Release Kinetics	Passive (P) or Triggered (T) Release	Ref.
***Growth factors***				
calcium phosphate ceramics	S	co-precipitation: 40%, adsorption: 80% (VEGF after 4 days in vitro)	P	[57]
PLGA scaffolds	S	unconjugated scaffold: 100% within 4 h, heparin-conjugated scaffold: 100% after 21 days (BMP-2 in vitro)	P	[78]
chitosan-silica membranes	S	hybrid membrane: 1.5 µg/mL, chitosan membrane: <0.5 µg/mL (BMP-2 in vitro)	P	[84]
gelatin hydrogel	S	reduced water content resulted in a longer BMP-2 retention in vivo	P	[120,121]
silk hydrogel	S	1% silk: 35%, 2% silk: 15% (BMP-2 day 1 in vitro)	P	[122]
PLGA-based fibrous scaffold	S	absorption: burst (80% BMP-2 within 1 week), encapsulation: sustained release (80% BMP-2 after 35 days in vitro)	P	[133]
nanofibrous membranes	S	500 pg/day BMP-2 release rate (in vitro)	P	[134]
core-shell microspheres	D	core: 80% within 24 days, shell: 80% within 6 days (BMP-2 and FGF-2 in vitro)	P	[170]
PLGA microspheres within porous PLGA cylinder	D	20% (BMP-2) or 10% (VEGF) remaining (14 days in vivo)	P	[171]
PLGA microspheres within gelatin hydrogel	D	microspheres: <20% BMP-2 after 30 days, hydrogel: >70% VEGF within 7 days (in vitro)	P	[173]
calcium phosphate scaffold loaded with nanocellulose	D	single drug carrier: 3.19% BMP-2 and 7.91% VEGF, dual drug carrier: 3.67% BMP-2 and 4.68% VEGF (day 1 in vitro)	P	[174]
PLGA nanoparticles and alginate microcapsules	D	sequential release: 100 ng within 4 days (BMP-2) and 14 days (VEGF) in vitro	P	[178]
PLA-coated implants	D	54% IGF-I and 48% TGF-β1 within 48 h (in vitro)	P	[183]
gelatin hydrogels	D	70–80% SDF-1 and 45–55% BMP-2 (day 1 in vivo)	P	[184]
silk microspheres within hydroxyapatite scaffold	D	BMP-2 adsorption: >80% within 7 days, BMP-2 encapsulation: >60% within 14 days (in vitro)	P	[185]
heparinized mineralized collagen type I matrix scaffolds	D	4–10% BMP-2 and ~0.5% SDF-1α of loaded growth factors released after 6 weeks in vitro	P	[186]
alginate fibers within PLA polymer	D	sequential release: 2500 pg/mL after 2–3 weeks (BMP-2) and 28 days (VEGF) in vitro	P	[202]
PEG-hydrogel	S	VEGF release and scaffold degradation within 2–3 days (50 µg/mL collagenase in vitro)	T	[216]
sono-disruptable liposomes	S	30 s: 5 µg/mL, 60 s: 7 µg/mL (BMP-2, 1 MPa, in vitro)	T	[234]
***Hormones***				
layered scaffold	S	daily pulsatile PTH release over 21 days (in vitro), 98.5% loading efficiency	P	[76,77]
thermo-sensitive liposomes	S	stimulus at day 3: >20%, stimulus at day 8: <10% (PTHrP, 42 °C in vitro)	T	[228]
***Bisphosphonates***				
collagen sponge	S	50% ibandronate after 50 h (in vitro)	P	[43]
calcium phosphate scaffolds	S	1 mg/scaffold: 31.33% ± 1.58%, 5 mg/scaffold: 7.99% ± 0.08% (alendronate, within 1 day in vitro), >72% loading efficiency	P	[65]
hydroxyapatite-coated titanium implants	S	burst release order: zoledronate > ibandronate > pamidronate (within 7 days in vitro)	P	[104]
PLA-calcium phosphate-coated magnesium-based alloys	S	14% within 3 days: diffusion, up to 27% within 4 weeks: degradation of implant coating (zoledronate, in vitro)	P	[110]
hydroxyapatite nanoparticles	S	>60% zoledronate after 1 h (in vitro), loading efficiency between 28.15 ± 4.78% and 52.14 ± 8.47%	P	[148]
hydroxyapatite-coated titanium implants	D	dual drug loading reduced initial burst compared to single drug coating by almost 40% at day 1 (zoledronate and bFGF, in vitro)	P	[190]
redox-sensitive nanofibers	S	~20% BMP-2 release by stepwise increase in glutathione concentration (in vitro)	T	[226]
***Glucocorticoids***				
nanoparticle-embedded electrospun nanofiber	D	BMP-2: 30% after 300 h, dexamethasone: 30% within 100 h (in vitro)	P	[194]
polypyrrole-filled electrically responsive microreservoires	S	~20% dexamethasone release by each stimulus (voltage cycles between −1 V and +1 V in vitro)	T	[208]
chitosan-functionalized mesoporous silica nanoparticles	D	pH 6.0: 80% after 50 min, pH 7.4: 10% after 80 min (dexamethasone, in vitro)	T	[227]
***NSAIDs***				
micelle-loaded titania nanotube arrays	D	sequential release due ratio of micelle (hydrophobic indomethacin) to inverted micelle (hydrophilic gentamicin) (in vitro)	T	[231]
***Prostaglandin E2 receptor agonist***				
PEG nanogel	D	~30% released within 30 min, ~70% remained for 7 days (prostaglandin E2 receptor agonist, in vitro)	P	[193]
***Enzyme inhibitors***				
micelles	S	>50% GSK3β inhibitor in 5 h (in plasma at 37 °C)	P	[165]
polyelectrolyte particulate coating	S	~50% bortezomib release at stimulus (42 °C in vitro)	T	[229]
***Sphingosine 1-phosphate receptor agonists***				
PLGA-coated allografts	S	0.57 mg FTY720 released in 14 days in vitro; 64% loading efficiency	P	[92]
polymeric microspheres	S	slow-degrading (more hydrophobic): >25%, fast-degrading: <10% (FTY720, after 20 min in vitro)	P	[154]
***Statins***				
calcium sulfate scaffolds	S	>70% BMP-2 after 14 days in vitro (higher loading reduced release rate)	P	[58]
polyurethane scaffolds	S	almost linear trend 200 µg/g foam: 20%, 20 µg/g foam: 10% (lovastatin, after 30 days in vitro)	P	[73]
PLGA membranes	S	1 µg/day release rate (fluvastatin, in vitro)	P	[75]
PCL nanofibers	S	absorption: burst, incorporation during fabrication: sustained release (simvastatin)	P	[85]
PLGA-PEG hydrogel	S	>50% simvastatin after 2 days (in vitro)	P	[115]
PLGA-hydroxyapatite microspheres	S	>20% simvastatin after 1 day (in vitro)	P	[151]
PEG-based micelles	S	60% atorvastatin after 10 h (in vitro)	P	[162]
***Metal ions***				
PLGA scaffold containing black phosphorus	S	10 s: 37%, 300 s: 45% (strontium, light irradiation)	T	[230]
***Antibiotics***				
calcium phosphate carrier	D	calcium-deficient hydroxyapatite: 70% loading (50% tetracycline release after 20 h), hydroxyapatite: 55% loading (50% tetracycline release in 5 h in vitro)	P	[199]
